# Hedgehog pathway maintains cell survival under stress conditions, and drives drug resistance in lung adenocarcinoma

**DOI:** 10.18632/oncotarget.8253

**Published:** 2016-03-22

**Authors:** Erh-Hsuan Lin, Yu-Rung Kao, Chih-An Lin, Ting-Yu Kuo, Sheng-Ping Yang, Chiung-Fang Hsu, Teh-Ying Chou, Chao-Chi Ho, Cheng-Wen Wu

**Affiliations:** ^1^ Institute of Biomedical Sciences, Academia Sinica, Taipei, Taiwan; ^2^ Institute of Microbiology and Immunology, National Yang Ming University, Taipei, Taiwan; ^3^ Institute of Clinical Medicine, National Yang-Ming University, Taipei, Taiwan; ^4^ Institute of Biochemistry and Molecular Biology, National Yang Ming University, Taipei, Taiwan; ^5^ Department of Clinical Laboratory Sciences and Medical Biotechnology, National Taiwan University Medical College, Taipei, Taiwan; ^6^ Department of Pathology and Laboratory Medicine, Taipei Veterans General Hospital, Taipei, Taiwan; ^7^ Department of Internal Medicine, National Taiwan University Hospital and National Taiwan University Medical College, Taipei, Taiwan

**Keywords:** lung adenocarcinoma, Hedgehog pathway, HHIP, drug resistance, HGF/MET

## Abstract

Hedgehog (HH) pathway plays an important role in embryonic development, but is largely inactive in adult except for tissue repair. Aberrant activation of HH pathway has been found in a variety of cancer types. In non-small cell lung cancer, however, the role and importance of HH pathway remain controversial. In the current study, we found that HH pathway was maintained in low activity in lung adenocarcinoma (LAC) cells under normal culture condition, but was highly induced in response to stress conditions. Activation of HH pathway promoted cell survival, growth, and invasion partially through HGF and MET signaling. Hedgehog-Interacting Protein (HHIP), a cell-surface negative regulator of HH pathway, was epigenetically silenced in LAC. Overexpression of HHIP blocked the activation of HH and HGF/MET pathways, and made cells significantly more susceptible to stress conditions. In LAC cells with acquired resistance to Epidermal Growth Factor Receptor Tyrosin Kinase Inhibitor (EGFR-TKI), we found that a part of tumor cells were much more sensitive to HH or HGF/MET inhibitors, suggesting an oncogenic addiction shift from EGFR to HH and HGF/MET pathways. In conclusion, this study showed that HH pathway is a survival signaling that drives LAC cell growth under stress conditions, and HHIP is a key regulator to block the induction of HH pathway. Targeting the HH pathway through inhibitors or HHIP thus holds promise to address EGFR-TKI resistance in LAC in clinic.

## INTRODUCTION

Lung cancer is the leading cause of cancer mortality worldwide. Non-small cell lung cancer (NSCLC) accounts for about 75% of lung cancers, among which the lung adenocarcinoma (LAC) is the most common histological subtype (about 40%). LAC is frequently associated with activating epidermal growth factor receptor (EGFR) mutations, and chemotherapy has limited efficacy [1–3]. Although the development of EGFR Tyrosine Kinase Inhibitors (TKIs) has offered an improved progression-free survival in LAC patients, drug resistance invariably occurred [1, 4]. In clinic, the T790M point mutation on EGFR represents approximately 50–60% of all recurrent lung tumors with acquired resistance to current 1st-generation EGFR-TKIs (Erlotinib and Gefitinib) [1, 4, 5]. Besides EGFR-T790M, HGF/MET signaling also plays an important role in EGFR-TKI resistance. MET amplification has been reported in around 4% of recurrent cases, which induces the resistance through ERBB3 (HER3)-dependent activation of PI3K [5, 6]. HGF-mediated MET activation has also been found to mediate intrinsic and acquired resistance to EGFR-TKI by restoring PI3K/AKT pathway independent of EGFR or ErbB3 [7]. Higher serum levels of HGF were significantly associated with shorter progression-free survival and overall survival in LAC patients receiving EGFR-TKI treatment [8].

Tumor cells are continually subjected to diverse stress conditions from microenvironment, such as hypoxia, nutrient deprivation, and oxidative, genotoxic, and replicative stresses [9, 10]. Stemness properties have been implicated in cell survival against stress conditions, and mediate tumor initiation, metastasis, and therapeutic resistance [11, 12]. Hedgehog (HH) pathway is a stemness pathway tightly regulated during development, and directs segments and organ formation. In adults, HH pathway is largely inactive, except for its function in tissue repair or maintenance [13]. However, aberrant activation of HH signaling has been identified in a variety of cancer types, driving proliferation, self-renewal and tumorigenesis in cancer stem-like cells [14]. HH pathway performed extensive crosstalk with other oncogenic or stemness signalings, such as RAS/RAF/MEK/ERK, PI3K/AKT, EGFR, and Notch [15]. In small-cell lung cancers (SCLCs), a subset of tumor samples were found to maintain their malignant phenotype through ligand-dependent HH pathway, and inhibition of HH pathway activity repressed tumor growth [16]. In NSCLC, the role of HH pathway remains controversial. It was initially suggested that HH pathway activity was low and cells were insensitive to HH inhibitor [16, 17]. However, other studies argued that a large percentage of NSCLC tumor samples showed high HH pathway activity, and a number of NSCLC cell lines were sensitive to HH inhibitors [18–21].

PTCH is the cell surface receptor of HH pathway, which positively regulates the downstream HH signaling through SMO. Hedgehog-interacting protein (HHIP) is a membrane glycoprotein that binds to all three mammalian HH ligands with an affinity comparable to PTCH [22, 23]. HHIP does not contain the intracellular domain, thus attenuates the HH pathway by competing HH ligands with PTCH [23]. HHIP and PTCH are both transcriptional targets of GLI1 after activation of HH pathway, and the HHIP-mediated negative regulatory feedback loop plays an important role in development such as lung branching morphogenesis [24]. HHIP expression has been found silenced by promoter hypermethylation in several types of tumor, including gastrointestinal [25], hepatocellular carcinoma [26, 27], medulloblastoma [28], and pancreatic neoplasm [29]. Nevertheless, how HHIP may regulate HH pathway and tumor progression has not been well studied in NSCLC.

## RESULTS

### The gene expression of HHIP is significantly reduced in LAC patient samples and cell lines

**Figure 1:**
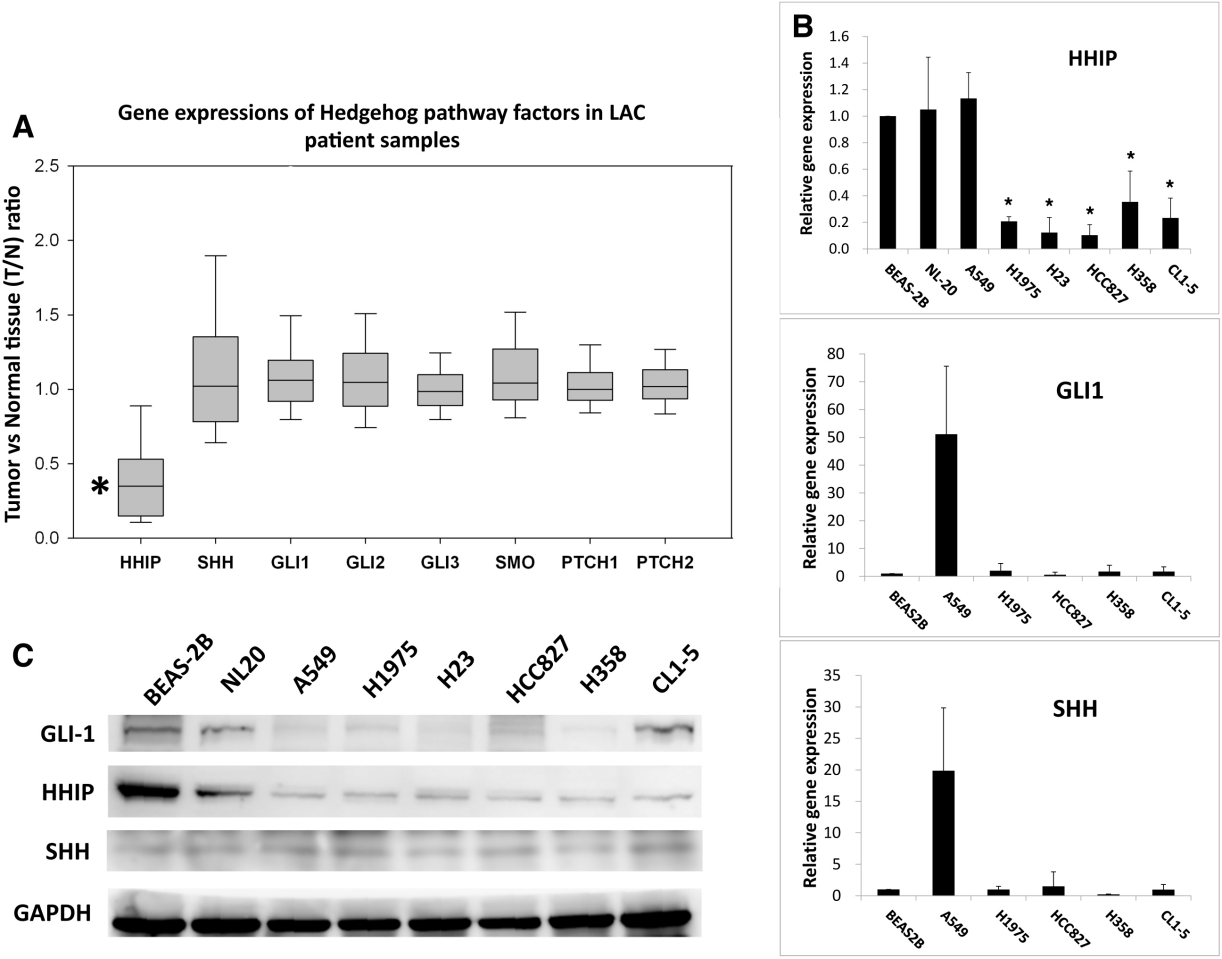
HHIP is significantly down-regulated in LAC patient samples and cell lines (**A**) Two GEO microarray data sets (GSE19804, GSE27262) containing information of both tumor and adjacent normal tissue from totally 85 LAC patient samples were analyzed and presented together for gene expressions of HH pathway components. T/N ratio, the ratio of gene expression in tumor vs. in normal part. (**B**) The relative gene expressions of HHIP, GLI1, and SHH in different LAC cell lines. The gene expression was normalized by 18S in respective cell lines, and then compared to that of non-tumor lung epithelial cell line BEAS-2B (which is set as 1). (**C**) The western-blot of GLI1 and HHIP proteins in LAC cell lines. Paired *T*-Test (A) and Independent-Samples *T*-Test (B) were used to evaluate the significance of differences among samples. *P < 0.05. *n* = 85 for (A) and *n* = 3 for (B).

We first investigated whether HH pathway is abnormally activated in LAC. Three microarray data sets from public domain Gene Expression Omnibus (GEO) containing the gene expression information of both tumor and adjacent normal parts in LAC patients (GSE19804, GSE27262, and GSE10072) were analyzed. Comparing the relative gene expressions between tumor and normal parts (the T/N ratio), only HHIP was significantly reduced, while other HH pathway components largely unchanged (T/N closed to 1) (Figure 1A). One microarray data set (GSE10072) did not contain HHIP probe because of the chip version (Affimatrix HG-U133A), but T/N ratios of other genes still showed similar results (Supplementary Figure S1). To confirm the results, we investigated the gene expressions of SHH, GLI1, and HHIP in LAC cell lines available in our lab. Likewise, the results showed that most LAC cell lines (except for A549) showed a significantly reduced HHIP, but comparable GLI1 and SHH mRNA levels as compared to non-tumor lung epithelial cells BEAS-2B or NL20 (Figure 1B). In protein level, all LAC cell lines showed low HHIP, and comparable or lower GLI1 expressions as compared to BEAS-2B or NL20 (Figure 1C). Together, these results suggested that in LAC, the expression of HHIP was significantly suppressed, while most other HH pathway factors largely unchanged.

### The gene expression of HHIP is epigenetically silenced in LAC

**Figure 2:**
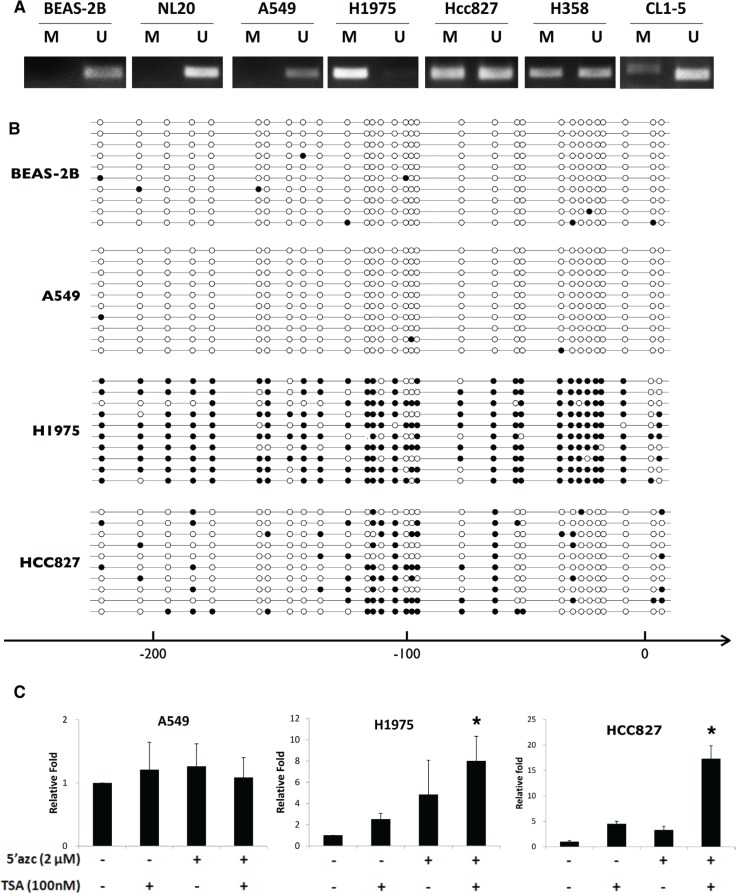
HHIP promoter is epigenetically silenced in LAC cells The methylation status of HHIP promoter in LAC cell lines were analyzed using (**A**) MSP and (**B**) BS (Supplementary Figure S2A). (**C**) The HHIP gene expression was analyzed in LAC cell lines after treatment with 5–-Azc (DNA methylation inhibitor) and TSA (histone acetylatransferase inhibitors). The solid circle indicates a methylated CG site, while empty circle unmethylated. Independent-Samples *T*-Test were used to evaluate the significance of differences among samples. *n* = 5 for (C).

It has been reported that HHIP was epigenetically silenced by promoter hypermethylation in different types of cancer [25–28]. We thus examined the methylation state of HHIP promoter in LAC. The results of methylation-specific PCR (MSP) confirmed that in most LAC cell lines (except for A549), HHIP promoter was intensively or partially methylated (Figure 2A and Supplementary Figure S2A). Four cell lines were further investigated by bisulfite sequencing (BS), and the results showed that the HHIP promoters in H1975 and HCC827 were hypermethylated, while BEAS-2B and A549 were not (Figure 2B and Supplementary Figure S2A). The treatment with 5–-Azc and TSA (the DNA methylation and histone acetylatransferase inhibitors, respectively) enhanced the HHIP expression in H1975 and HCC827, but not A549 cells (Figure 2C). To further confirm the methylation status of HHIP promoter in LAC, 492 patient samples from TCGA open data base were analyzed. The results showed that HHIP promoter was significantly hypermethylated in tumor as compared to normal tissue (Supplementary Figure S2B), and the methylation was significantly associated with HHIP gene expression (Supplementary Figure S2C).

### HHIP overexpression significantly inhibited LAC cell proliferation, clonogenicity, invasion, and spheroid formation in serum-starvation state

**Figure 3:**
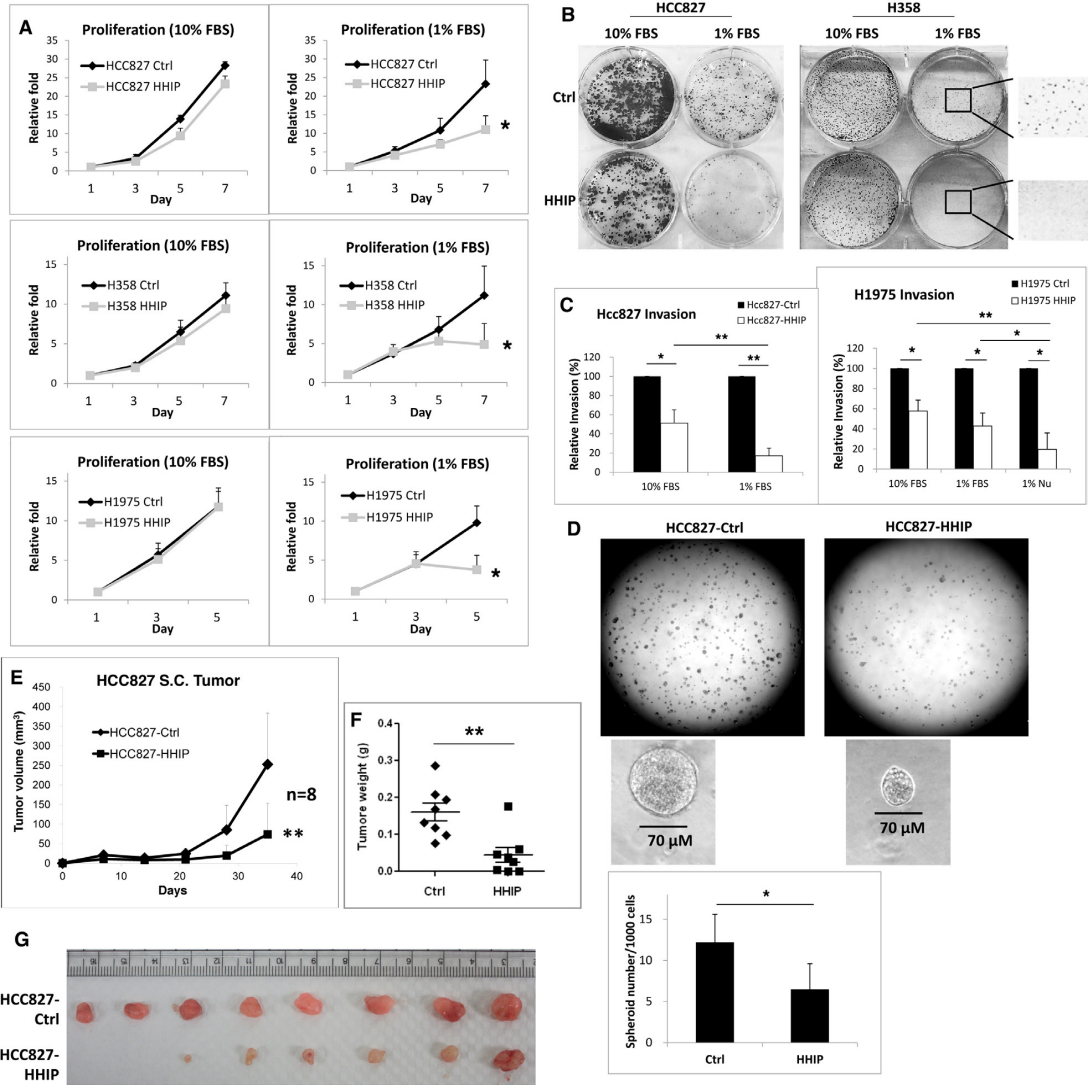
HHIP overexpression significantly inhibited cell proliferation, clonogenicity, invasion, and tumor spheroid formation in serum-starvation state LAC cell lines overexpressing HHIP or RFP as control protein (Ctrl) were analyzed for their (**A**) proliferation rate, (**B**)^#^ clonogenicity in 2D culture dish, (**C**) invasion activity in matrigel-coated transwell, in mediums containing 10% FBS, 1% FBS, or 1% Nu-serum. (**D**) The tumor spheroid formation analysis was performed by seeding HCC827 cells in serum-free matrigel. For tumor formation analysis, 1 × 10^6^ HCC827 cells were implanted subcutaneously in nude mice, and measured for (**E**) tumor size, and (**F**) tumor weight after sacrificed on day 35. (**G**) The photo of resected tumors. Independent-Samples *T*-Test were used to evaluate the significance of differences among samples. *P < 0.05, **P < 0.01. *n* = 3 for (A) and (C), *n* = 6 for (D), *n* = 8 for (E–G). #H358 generally formed smaller colonies in 1% FBS. For a clear vision, the full-size original image of H358 colonies was provided in Supplementary Figure S3.

We then investigated the role of HHIP silencing in LAC. HHIP or Red-Fluorescent Protein (RFP, as control protein) was overexpressed in 3 different LAC cell lines. Unexpectedly, HHIP overexpression only slightly reduced cell proliferation and clonogenicity in LAC cells under normal culture condition (10% FBS) (Figure 3A and 3B). However, when cells were cultured in serum-starvation state (1% FBS), HHIP overexpression significantly inhibited cell proliferation and clonogenicity (Figure 3A and 3B, and Supplementary Figure S3 for the full-size images of colonies). Likewise, HHIP overexpression inhibited cell invasion more significantly in serum-starvation state in 1% FBS or 1% Nu-serum (a low-protein cell growth supplement) (Figure 3C). Finally we tested the importance of HHIP in spheroid formation in serum-free 3D matrix. The results showed that cells overexpressing HHIP formed significantly less spheroids (Figure 3D). Together, these data suggested that although the silencing of HHIP may not significantly influence cell functions under normal culture condition, it plays an important role to maintain cell proliferation, invasion, survival, and spheroid formation under serum-starvation state.

### LAC cells overexpressing HHIP showed defective tumor formation and growth activities *in vivo*

To verify whether HHIP influences *in vivo* tumor formation and the growth of LAC cells, we implanted LAC cells overexpressing HHIP or RFP subcutaneously in nude mice. The tumor growth was followed for 1 month. The result showed that tumors overexpressing HHIP had a 70% reduced average volume and 63% reduced average weight as compared to control tumors (Figure 3E and 3F). Cells overexpressing HHIP did not form tumor in 2 of the 8 implanted mice, while control cells formed tumors in all mice (Figure 3G). The HHIP overexpression in tumors was confirmed using Western-Blot (Supplementary Figure S4). These results indicated that HHIP overexpression in LAC cells led to defective *in vivo* tumor formation and growth.

### HH pathway was activated in serum starvation state, and mediated HGF expression and MET phosphorylation in LAC cells

**Figure 4:**
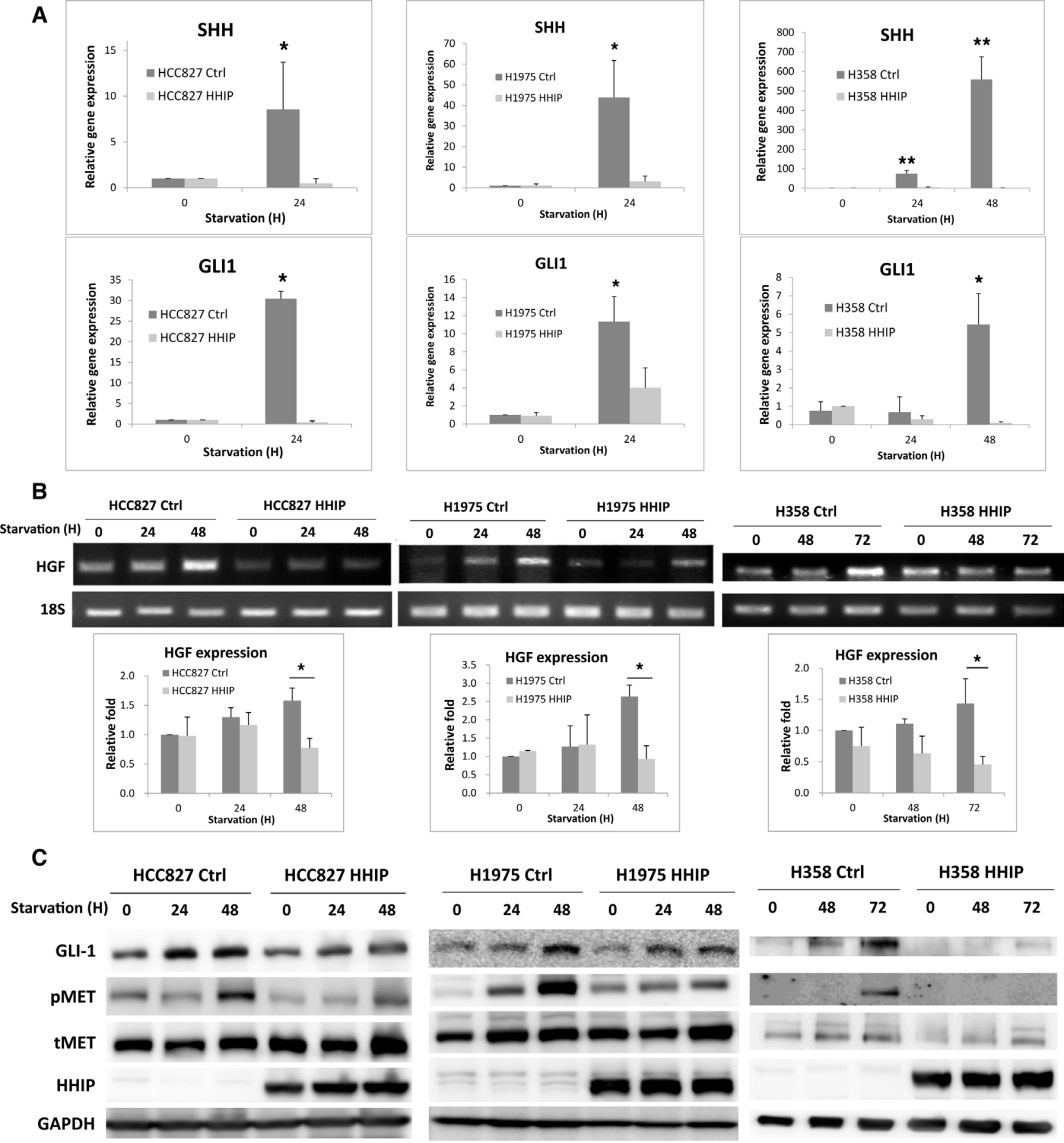
Hedgehog pathway was activated in serum-starvation state, which induced HGF expression and MET phosphorylation in LAC cells, while HHIP overexpression blocked such inductions (**A**) Gene expressions of GLI1 and SHH were measured using qPCR 24 or 48 hrs after serum-starvation (1% FBS) in LAC cells overexpressing HHIP or RFP (Ctrl). (**B**) HGF gene expression was detected using end-point PCR in LAC HHIP/Ctrl cells at indicated time points after serum-starvation (1% FBS), and quantified. (**C**) protein levels of pMET, MET, and GLI1 were detected using western-blot in LAC HHIP/Ctrl cells at indicated time points after serum-starvation (1% FBS). Independent-Samples *T*-Test were used to evaluate the significance of differences among samples. *P < 0.05, **P < 0.01. *n* = 3 for (A) and (B).

To clarify why HHIP plays a much more important role in serum-starvation state, we examined the activity of HH pathway. The gene expressions of GLI1 and SHH (the main transcription factor and ligand, respectively) were detected as indication for the pathway activity. The results showed that expressions of both genes were significantly induced within 2 days after serum-starvation (Figure 4A). Importantly, overexpression of HHIP blocked the inductions. To find the downstream oncogenic signaling, the expressions of several growth factors that have been indicated to interact with HH pathway were detected. The results showed that HGF was the most dominantly induced factor after SHH treatment or serum-starvation (Supplementary Figure S5A and Supplementary Figure S5B). Because gene expression analysis of HGF using standard quantitative PCR procedure frequently produced instable results, presumably due to the presence non-specific products (Supplementary Figure S5C), we then used end-point PCR to verify HGF gene expression. We observed that HGF was significantly induced within 72 hr after serum-starvation (Figure 4B). The protein level of GLI1 and phospho-MET (pMET) were also enhanced after serum-starvation (Figure 4C). Notably, overexpression of HHIP blocked these inductions (Figure 4B and 4C), suggesting that HGF/MET signaling was downstream of HH pathway. Similarly, knockdown of GLI1 reduced endogenous level of pMET (Supplementary Figure S5E). Serum-starvation or SHH treatment induced the expression of GLI1, but not HHIP, confirming that the epigenetic silencing of HHIP prevented its expression after HH pathway activation (Figure 4C and Supplementary Figure S5D). Taken together, these combined results indicated that under stress conditions such as serum-starvation, HH pathway was activated, which then induced the HGF expression and MET phosphorylation. Overexpression of HHIP blocked such inductions, suggesting that the silencing of HHIP was a critical step to potentiate HH activation and to maintain cell survival under stress conditions.

### HGF recovered the clonogenicity and invasion activity blocked by HHIP in serum starvation state

**Figure 5:**
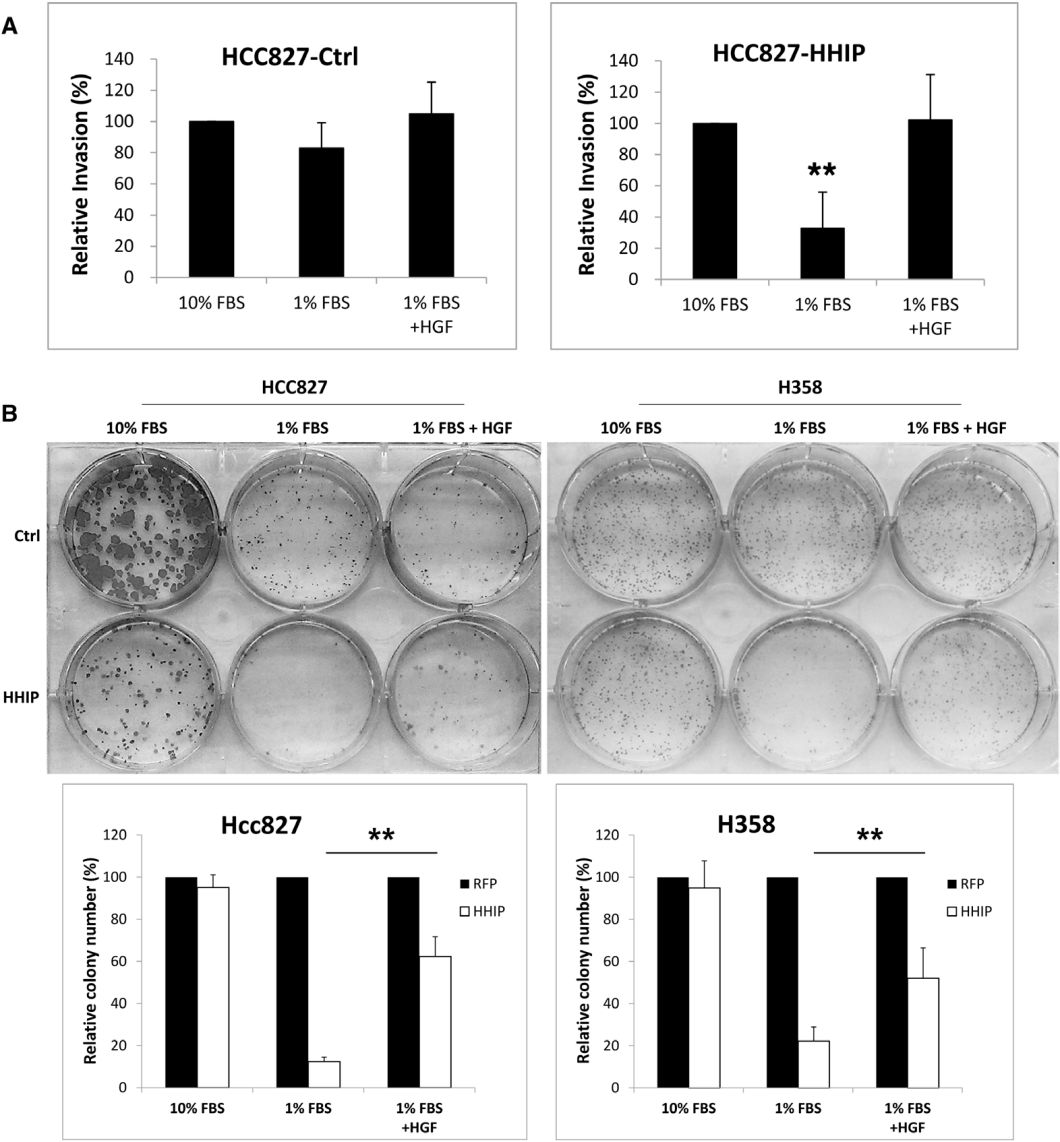
HGF treatment recovered the clonogenicity and invasion activities that were suppressed in LAC cells overexpressing HHIP in serum-starvation state (**A**) The invasion activity of HCC827 and (**B**) the clonogenicity of HCC827 and H358 cells overexpressing HHIP or RFP (Ctrl) were tested. Cells were cultured in the medium containing 10% FBS, 1% FBS, or 1% Nu-serum, with or without addition of HGF (20 ng/ml). Error bars indicate SD. Independent-Samples *T*-Test were used to evaluate the significance of differences among samples. *P < 0.05, **P < 0.01. *n* = 5 for (A), *n* = 3 for (B).

We then tried to verify whether HGF/MET plays an important role downstream of HH pathway. The recombinant HGF was added into cells cultured in mediums containing 10% or 1% FBS. The results showed that HGF recovered the invasion activity that was reduced in serum-starvation state in cells overexpressing HHIP, but showed minimal effect in control cells (Figure 5A). HGF also partially recovered the clonogenicity that was lost in serum-starvation state in cells overexpressing HHIP, but showed little effect in control cells (Figure 5B, and Supplementary Figure S6 for full-size images). However, HGF was not able to recover the sphere forming ability in HCC827 cells overexpressing HHIP (data not shown).

### Blocking HH pathway enhanced the sensitivity of LAC cells to EGFR-TKI

**Figure 6:**
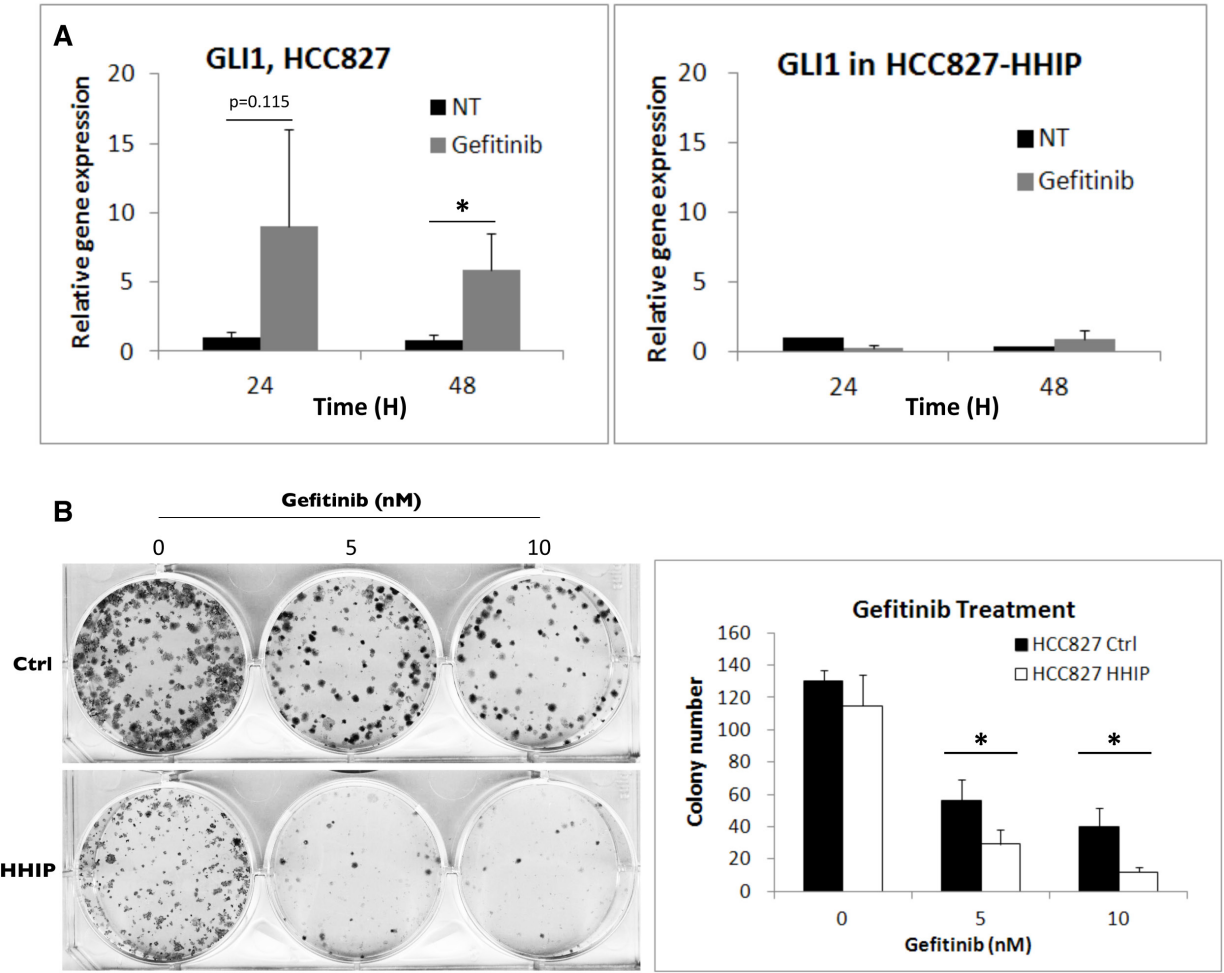
HH pathway was induced in LAC cells when treated with EGFR-TKI, while HHIP blocked such induction and further sensitized cells to EGFR-TKI treatment (**A**) The GLI1 gene expression was detected using qPCR at indicated time points after EGFR-TKI (Gefitinib, 100 nM) treatment in HCC827 cells overexpressing HHIP or RFP (Ctrl). (**B**) The clonogenicity of HCC827 HHIP/Ctrl cells was analyzed in 2D culture dish with the treatment of Gefitinib at indicated concentrations, and quantified. All cells were cultured in medium contain 10% FBS. Error bars indicate SD. Independent-Samples *T*-Test were used to evaluate the significance of differences among samples. *P < 0.05. *n* = 3 for (A), *n* = 4 for (B).

Since HH pathway can be activated in serum-starvation state to improve cell survival, we asked whether it can be also activated in response to drug treatment. LAC cells were treated with EGFR-TKI (Gefitinib), and the result showed that within 48 hr, the surviving cells showed enhanced GLI1 expression (Figure 6A). Once more, overexpression of HHIP blocked the induction. The clonogenicity was also tested, and the result showed HHIP overexpression significantly reduced the colony numbers when LAC cells were treated with EGFR-TKI (Figure 6B). These results indicated that HH pathway can be activated and improve cell survival in the treatment of EGFR-TKI.

### HH pathway maintained cell survival and growth in LAC cells with acquired resistance to EGFR-TKI

**Figure 7:**
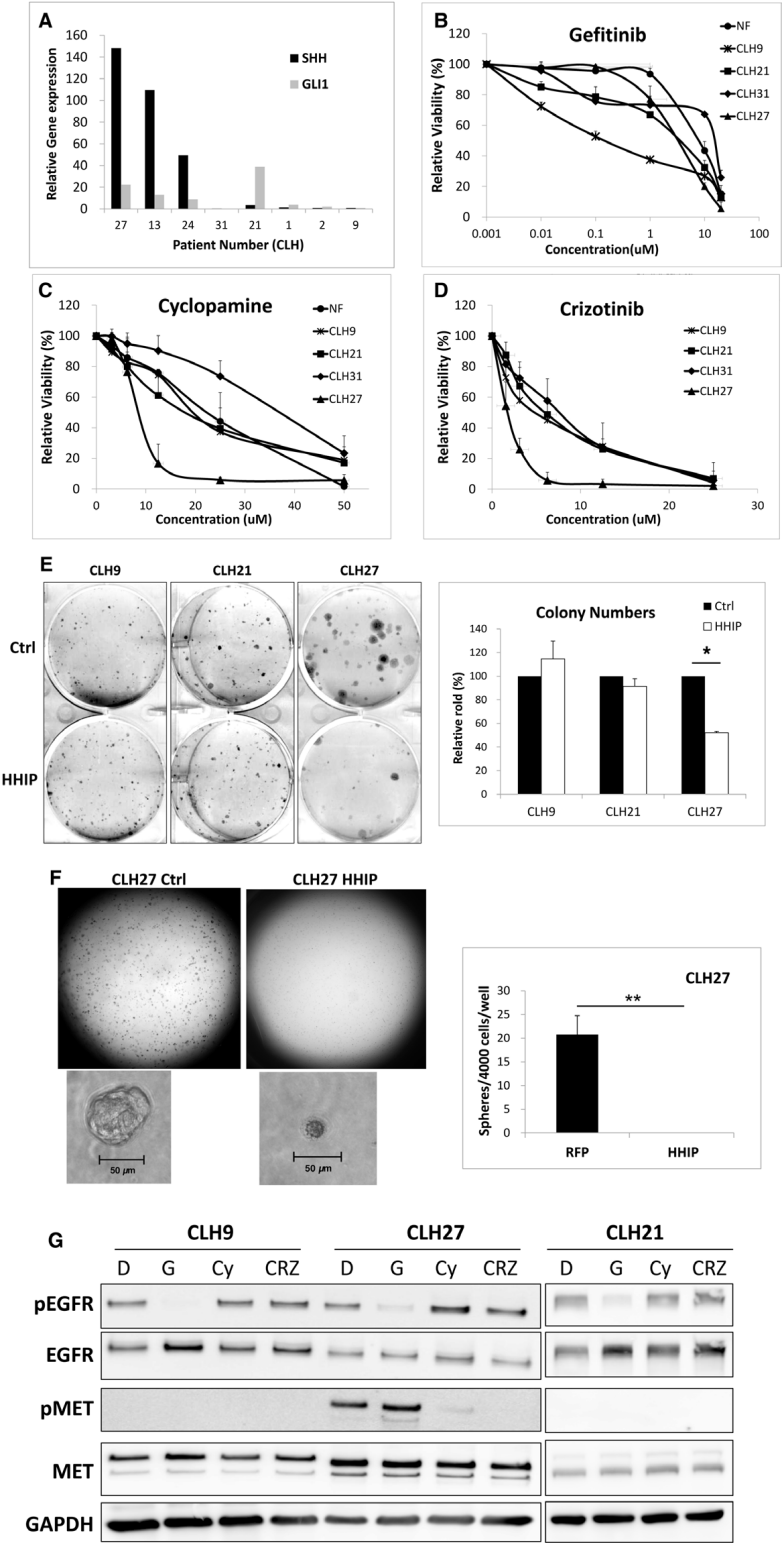
HH pathway maintained cell survival and growth in LAC cells with acquired resistance to EGFR-TKI through HGF/MET (**A**) LAC tumor samples obtained from patients relapsed from EGFR-TKI treatment (Supplementary Table S1) were analyzed for GLI1 and SHH gene expressions using qPCR. The gene expressions were normalized by 18S in respective samples, and then compared to a patient sample untreated for EGFR-TKI (CLH9). Primary cultured normal fibroblast (NF) and tumor cells were analyzed for survival curve against (**B**) Gefitinib, (**C**) Cyclopamine, or (**D**) Crizotinib treatment. (**E**) Primary cultured tumor cells overexpressing HHIP or RFP (Ctrl) were analyzed for the clonogenicity in 2D culture dish. (**F**) CLH27 cells overexpressing HHIP or RFP were analyzed for spheroid formation in serum-free 3D matrix, and the spheroid numbers were quantified. (**G**) Three primary cultured tumor cells were analyzed for EGFR and MET phosphorylation levels against different drug treatments using Western-Blot. D: DMSO, G: Gefitinib, Cy: Cyclopamine, Crz: Crizotinib. *P < 0.05. **P < 0.01. *n* = 3 for (B), (C), (D), and (E). *n* = 6 for (F). All cells were cultured in medium contain 10% FBS, except for (F).

Finally, we investigated the role of HH pathway in LAC cells with acquired resistance to EGFR-TKI. Primary lung tumor cells isolated from patients relapsed from EGFR-TKI treatment (Supplementary Table S1) were analyzed for HH gene expressions. The results showed that among the five relapsed samples (CLH27, 13, 24, 31, and 21), three showed both much higher SHH and GLI1 gene expressions (CLH27, 13, and 24), and one showed only high GLI1 expression (CLH21), as compared to the samples untreated (CLH9) or irresponsive to EGFR-TKI (CLH1 and 2) (Figure 7A and Supplementary Figure S7C). Like previous results, HHIP gene expression was reduced in these tumor cells as compared to normal fibroblast (NF) obtained from normal tissue of patients (Supplementary Figure S7A and Supplementary Figure S7B). Some tumor cells that can be stably cultured were tested for sensitivities to drugs. As expected, all cells showed low sensitivity to Gefitinib except CLH9 (Figure 7B). In contrast, cells from CLH27 were much more sensitive to Cyclopamine (an HH inhibitor) or Crizotinib (an MET inhibitor) comparing to others (Figure 7C and 7D). Overexpression of HHIP significantly suppressed the clonogenicity of CLH27 but not others, even in normal culture condition (10% FBS) (Figure 7E). In serum-free 3D matrix, HHIP overexpression significantly inhibited spheroid formation in CLH9 and 27 cells (Figure 7F and Supplementary Figure S7H). Similar results were observed in our laboratory-generated HCC827 cell lines with acquired resistant to EGFR-TKI (Supplementary Figure S7D–S7F), showing that resistant cells were more sensitive to Cyclopamine or Crizotinib as compared to parental cells. Finally, western-blot analysis confirmed that CLH27 cells showed higher MET phosphorylation level, which can be inhibited by Cyclopamine (Figure 7F), confirming that HGF/MET is a downstream of HH pathway in these cells. All together, these combined results showed that a part of LAC cells with acquired resistance to EGFR-TKI showed higher HH pathway activity, and were more sensitive to HH and HGF/MET inhibitors. Finally, methylation status of HHIP promoter in primary cell lines were analyzed. Unexpectedly, except for CLH9 (patient untreated for EGFR-TKI), cells from recurrent tumors showed little methylation on HHIP promoter, as analyzed by MSP or BS (Supplementary Figure S8). These results suggested that mechanism(s) other than DNA methylation still exists to suppress HHIP expression (Supplementary Figure S7A and S7B).

## DISCUSSION

In previous studies, controversial conclusions have been made concerning the role of HH pathway in NSCLC. It was initially identified in 2003 that ligand-dependent HH pathway maintained the malignant phenotype of a subset of SCLC, but was low or inactive in NSCLC [16]. A recent study also showed that HH pathway components, including SHH, PTCH, SMO, and GLI1 showed negative to weak expressions, and have no correlation with clinical outcomes [17]. In contrast, some studies reported that GLI1 and SHH can be positively stained in a large percentage of NSCLC samples (> 85%), and a subset of NSCLC cell lines were sensitive to HH inhibitors [18, 19]. The clinical outcome also showed contradictory results concerning the overall and progression-free survival in patients with high HH activity [21, 30]. These studies mainly relied on the immunohistochemical staining on SHH and GLI1 in tumor tissue arrays as indication of HH activity. Besides the different antibodies and staining procedures used that may raise inconsistent results, the simultaneous staining of paired tumor and adjacent normal tissue was absent. Only a small number of normal tissues were investigated in 2 studies (5 normal vs. 115 tumors [18], and 20 normal vs. 87 tumor [19] samples).

To approach the problem, we first compared the gene expression of HH pathway components between tumor and paired normal tissues. The analysis of 3 microarray data sets (GSE19804, GSE27262, and GSE10072) showed that all HH components, except for HHIP, had an average T/N ratio close to 1 (Figure 1A and Supplementary Figure S1). *In vitro* analysis showed that most LAC cell lines did not express significantly enhanced SHH or GLI1 than non-tumor lung epithelial cells, but had a remarkably reduced HHIP expression (Figure 1B and 1C). These results support the previous studies showing that HH pathway was weak or inactive in most LAC tumors [16, 17]. Nevertheless, our data showed that the negative regulator of HH pathway, HHIP, was aberrantly suppressed.

We then found that HHIP promoter was epigenetically silenced in LAC, like previous reports in other cancer types [25–29], The analysis of 492 LAC patient samples showed that HHIP promoter was significantly hypermethylated in tumor, and the methylation level was significantly associated with gene expression (Supplementary Figure S2B and Supplementary Figure S2C). BS and MSP analyses in LAC cell lines showed similar results (Figure 2). The only exception was A549, which showed simultaneously high levels of HHIP, GLI1, and SHH mRNA (Figure 1B). In accordance, HHIP promoter in A549 was little methylated, and the HHIP expression did not respond to TSA/5’Azc treatment (Figure 2). However, in protein level of GLI1 and HHIP, A549 showed the same results with other LAC cell lines (Figure 1C). This exception suggested that mechanism(s) other than promoter methylation and gene expression still exists to suppress HHIP protein expression in LAC.

We then tried to Figure out why the down-regulation of HHIP in LAC cells was not accompanied by up-regulations of GLI1 and SHH. Since stemness pathways have been implicated in cell survival against stress conditions instead of keeping active [11, 12], we hypothesized that HH pathway could be conditionally activated, and the silencing of HHIP potentiates such induction. To test the hypothesis, we investigated the outcome of HHIP overexpression in LAC cells in serum-starvation state. We found that while HHIP overexpression did not significantly inhibit cell functions, such as proliferation, clonogenicity, and invasion under normal culture condition (10% FBS), it did when cells were cultured in serum-starvation state (1% FBS or 1% Nu-serum) (Figure 3A–3C). LAC cells overexpressing HHIP formed much less spheroids in serum-free 3D matrix (Figure 3D, 7F and Supplementary Figure S7H). The formation of non-adherent sphere is a widely-applied laboratory method to evaluate stemness activity for both normal and cancer cells [31]. Inhibition of spheroid formation by HHIP suggested that HH pathway was activated and drove cell survive and self-renewal in stress condition. Similar outcome was shown in subcutaneous tumor formation analysis *in vivo*, an avascular environment short of serum support. We found that cells overexpressing HHIP exhibited defective tumor initiation and growth activity in nude mice (Figure 3E–3G).

Therefore, the combined data above suggest that in LAC cells, HH pathway serves as a survival signaling, which is inactive or kept in low level under normal condition, but is activated in stress conditions to maintain cell survival, proliferation, and self-renewal. In normal cells, HHIP expression can be induced after the activation of HH pathway because HHIP is a transcription target of GLI1, and HHIP-mediated negative regulatory feedback loop plays an important role in development [24]. In lung cancer, however, the expression of HHIP cannot be induced because of epigenetic silencing (Figures 2 and 4C and Supplementary Figure S4D), which leads to an aberrantly activated HH pathway. The silencing of HHIP in LAC thus plays an important role to potentiate the activation of HH pathway under stress conditions. These findings could also explain why past studies using GLI1 or SHH as biomarkers made controversial conclusions for the importance of HH pathway in NSCLC [16–21, 30], because the two factors are conditionally, but not constitutively activated.

We also defined that HGF/MET signaling is an important downstream of HH pathway. HGF gene expression and MET phosphorylation can be induced in response to SHH treatment or serum-starvation (Figure 4B and 4C, Supplementary Figure S5A–S5C), while overexpression of HHIP blocked such inductions. Knockdown of GLI1 also reduced the endogenous level of phosphor-MET (Supplementary Figure S5E). Unexpectedly, although addition of recombinant HGF recovered the invasion and (partially) 2D colony forming ability of LAC cells overexpressing HHIP in serum-starvation state (Figure 5), it cannot recover the spheroid-forming ability inhibited by HHIP (data not shown). These results suggest that besides HGF/MET, HH pathway still drives other oncogenic signalings to maintain cell survival and self-renewal in stress conditions, which deserve further studies. The interaction between HH and HGF/MET signalings has also been reported in other cell type. Lim et al. showed that HH pathway reduced HGF level in stromal cells during prostate branching morphogenesis, in contrast to our finding [32]. This difference reflects that the function of HH pathway can be diverse in different cell types, which further complicate its role in microenvironment *in vivo*.

Serum-starvation is a general laboratory procedure to mediate stress conditions, which can induce nutrient deprivation, hypoxia, or autophagy in cancer cells [33]. However, serum-starvation is hard to link directly to a single specific physiological condition. We thus tested whether EGFR-TKI treatment could also generate a stress condition and induce HH and HGF/MET signalings. The results confirmed that although a lot of LAC cells died after EGFR-TKI treatment within 48 hr (data not shown), GLI1 expression was induced in surviving cells, and once more, the overexpression of HHIP blocked such induction (Figure 6A). Overexpression of HHIP made cells significantly more susceptible to EGFR-TKI treatment (Figure 6B). We then investigated the importance of HH pathway in LAC cells with acquired resistance to EGFR-TKI, a major problem of LAC treatment in clinic. In both laboratory-established and clinically obtained LAC cells stably resistant to EGFR-TKI, a tendency of growth dependence on HH pathway was observed (Figure 7, Supplementary Figure S7 and Supplementary Table S1). Three of the 5 EGFR-TKI resistant primary LAC tumors showed higher expressions of both SHH and GLI1 as compared to tumors untreated or irresponsive to EGFR-TKI (Figure 7A and Supplementary Table S1). Primary cells of CLH27 were much more susceptible to Cyclopamine, Crizotinib, or HHIP overexpression (Figure 7C–7F). CLH27 showed high pMET level, which can be inhibited by Cyclopamine, confirming that HH is a upstream of HGF/MET in these cells (Figure 7G). Cells from CLH21, a relapsed LAC tumor harboring EGFR-T790M mutation, were not sensitive to Cyclopamine or HHIP overexpression, presumably due to the T790M-driven EGFR activation that maintained cell survival (Supplementary Table S1 and Figure 7C–7E). CLH21 showed only high expression of GLI1 but not SHH (Figure 7A and Supplementary Figure S7C), suggesting that GLI1 might be induced through non-canonical HH pathway. Whether GLI1 plays a role in T790M-driven EGFR-TKI resistance remains to be investigated. In summary, these combined data would suggest that a part of tumor cells have activated HH pathways to survive against EGFR-TKI treatment, and then shifted their oncogenic addiction to HH pathway for a long-term maintenance of cell growth. Finally, the methylation status of HHIP promoter in EGFR-TKI resistant cells were investigated. Unexpectedly, primary cell lines with acquired resistance to TKI (CLH13, 21, 24, 27, 31) showed little methylation on HHIP promoter (Supplementary Figure S8), while the HHIP expression was still repressed in both mRNA and protein levels (Supplementary Figure S7A and Supplementary Figure S7B). These results suggest that promoter methylation was not the only mechanism to repress HHIP expression. How EGFR-TKI resistant cells down-regulate HHIP and maintain HH pathway activity deserves further dissection.

In conclusion, our current study demonstrates the importance of HH pathway in LAC. HH pathway is inactive or kept in low level under normal condition, but is highly induced in response to stress conditions and maintains cell survival. The silencing of HHIP is required for the induction of HH and HGF/MET pathways. Overexpression of HHIP makes LAC cells much more susceptible to stress conditions. In LAC cells with acquired resistance to EGFR-TKI, a part of cells may shift their oncogene addiction from EGFR to HH pathway. Targeting HH pathway through inhibitors or HHIP recombinant protein thus holds promise to address EGFR-TKI resistance of LAC. Future studies will focus on how HH pathway is induced in response to stress, and how it maintains the oncogenic phenotype of LAC cells other than HGF/MET signaling. Finally, because of the limitation in the availability of EGFR-TKI resistant LAC tumors from relapsed patients, only a small number of samples were tested, and only 4 primary tumor cells can be adapted to stable culture and drug test. More samples will be necessary to confirm the clinical relevance of HH pathway in EGFR-TKI resistance.

## MATERIALS AND METHODS

### Cell culture

Human LAC cell lines A549 (ATCC CCL-185), H1975 (ATCC CRL-5908), HCC827 (ATCC CRL-2868), and H358 (ATCC CRL-5807) were cultured in RPMI medium containing 10% fetal bovine serum as previously described [34]. Growth factors HGF (R & D Systems), and anti-cancer drugs Gefitinib (Cell Signaling), Crizotinib (Cell Signaling), and Cyclopamine (Santa Cruz) were added to the culture medium in conditions as indicated in Figure legends.

### Primary cultures of tumor cells from LAC patients

EGFR-TKI resistant LAC cells were harvested from malignant pleural effusion of patients who were refractory to EGFR-TKI at the National Taiwan University Hospital (NTUH) (Supplementary Table S1). All cells in pleural effusion were collected by centrifuge, and RBCs were removed using RBC lysis buffer. After wash and centrifuge, the cells were seeded in 12-well plates (5 × 10^5^ cells/well) at 37°C in a humidified atmosphere containing 20% O_2_ and 5%CO_2_. Serum-free culture medium was used to prevent fibroblast overgrowth. After 12~24 hours, suspension cells in the wells were collected and cultured in a new plate with RPMI medium containing 10% FBS.

### Plasmids

Lentiviral vectors pLAS3w.RFP-C.Ppuro and pLAS3w.Ppuro were purchased from National RNAi Core Facility Platform (Academia Sinica). HHIP and GLI1 cDNA (library NIH_MGC_17) were obtained from VYM Genome Research Center (National Yang-Ming University). HHIP and GLI1 cDNAs were amplified by PCR and ligated into pLAS3w.Ppuro via NheI, EcoRV, and PstI sites downstream of CAG promoter to generate pLAS3w.HHIP.Ppuro and pLAS3w.GLI1. Ppuro expression vectors, respectively. pLKO.1-shGLI1 (clone ID: TRCN0000020485) that expresses the shRNA against GLI1 was obtained from the National RNAi Core Facility, Academia Sinica (Taipei, Taiwan), with the target sequence: CCTGATTATCTTCCTTCAGAA.

### Bisulfite sequencing (BS) and methylation-specific PCR (MSP) analysis of HHIP promoter

BS-specific primers (HHIP-BS-F and HHIP-BS-R) and BS process were performed according a previous publication [35] (Supplementary Figure S2, Supplementary Table S2). Sequencing of CT converted HHIP promoters was performed in IBMS Core Facility (Academia Sinica). The methylation state was analyzed using BiQ Analyzer (http://biq-analyzer.bioinf.mpi-inf.mpg.de, Max Planck Institut Informatik, Germany). MSP was performed according to a previous report [36] (Supplementary Figure S2), and the primers (HHIP-MSP-Meth-F, HHIP-MSP-Meth-R, HHIP-MSP-nonMeth-F, and HHIP-MSP-nonMeth-R) are listed in Supplementary Table S2.

### Reverse transcription-PCR

Quantitative PCR was performed as previously described [37], and the primers used are listed on Supplementary Table S2. For detection of HGF gene expression, end-point PCR and gel electrophorsis were performed. RNA was reverse transcribed using the gene specific primer HGF-1259R, and the cDNA was subjected to PCR using the primer pair HGF-739F and HGF-964R. For internal control, the same RNA sample was reversed transcribed using random hexamer, and 18S was measured using the primer 18S-F and 18S-R. The thermo-cycle program is listed on Supplementary Table S3.

### Western-blotting

Western-Blotting was performed as previously described [37]. Primary Antibodies included Anti-GLI1 (Rabbit monoclonal, Abcam), Anti-HHIP (Rabbit polyclonal, Genetex), Anti-β-tubulin (Mouse monoclonal, Enogene), Anti-Met (Rabbit monoclonal, Cell Signaling Technology), and Anti-phospho-Met (Rabbit monoclonal, Cell Signaling Technology). Secondary antibodies included Anti-rabbit IgG (Goat polyclonal, Peroxidase conjugated, Merck Millipore) and Anti-mouse IgG (Goat polyclonal, Peroxidase conjugated, Merck Millipore).

### 2D colony formation assay

HCC827 (2000 cells/well) or H358 (5000 cells/well) cells were seeded in 6-well plates in RPMI medium containing 10%, or 1% FBS as indicated on the figures, for 20–24 days without replenishing medium. HCC827 cells cultured in medium containing 10% FBS were cultured for only 10–14 days, preventing the overgrowth of colonies (Supplementary Figure S6B). For the experiment evaluating the effect of HGF (Figure 5), the medium containing 0 or 20 ng/ml of HGF was refreshed every 3 or 4 days. At the end of experiment, cells were washed twice with PBS and fixed with 4% paraformaldehyde for 15 min, and then stained with 0.1% crystal violet for 30 min, and de-stained with distilled water.

### *In vitro* tumor spheroid formation assay

Before experiment, 96-well plates were coated with 0.7% agarose (50 μl/well) and incubated at room temperature for more than 1 hr. HCC827 HHIP/Ctrl cells were suspended in Matrigel (BD Biosciences) and loaded on the top of agarose layer (2000 cells/30 μl/well), incubated at room temperature for more than 30 min. To avoid pipetting error, mixtures for 6 replicates were prepared in one tube, and loaded to 5 wells. DMEM/F12 medium containing 1 × N-2 Supplement (Thermo Fisher Scientific Inc.), 50 ng/ml of EGF, and 20 ng/ml of FGF was added to wells (200 μl/well), and refreshed every 3 or 4 days. Cells were cultured for 14 days. The spheroids were examined and imaged with a ZEISS Observer A1 microscopy under 2.5X and 10X object lens.

### Invasion assay

Non-transparent trans-wells (BD Biosciences) were coated with 7 μl of matrigel (BD Biosciences). 2 × 10^4^ cells were suspended in 200 μl of RPMI containing 10% FBS, 1% FBS or 1% Nu-serum (a low-protein cell growth supplement, BD Biosciences), and seeded on matrigel. Eighteen hr later, cells were fixed with methanol and stained with Hoechst (Invitrogen). The invasion cells were imaged using fluorescent microscopy and quantified with ImageJ software.

### *In vivo* tumor formation assay

Five-week-old nude mice (BALB/cAnN.Cg-Foxn1 nu/CrlNarl) were purchased from National Laboratory Animal Center (Taipei, Taiwan), and maintained in Taiwan Mouse Clinic (IBMS, Academia Sinica). HCC827 cells overexpressing HHIP or RFP (as control protein) were subcutaneously injected into the flank region of mice (1 × 10^6^ cells/mouse). Tumor sizes were measured weekly with a dial caliper. All mice were sacrificed 5 weeks after injection, and the tumors were resected, weighed, and photographed.

### Microarray data analysis

The microarray data sets GSE19804, GSE27262, and GSE10072 were retrieved from the public domain of Gene Expression Omnibus on National Center for Biotechnology Information (GEO, NCBI). The expression levels of HH pathway component genes between each tumor and the paired normal tissue were compared and presented as T/N ratios (gene expression level in tumor divided by that in normal tissue) on the figures.

### Study approval

For primary culture of LAC tumor cells, the samples were procured and utilized according to approved IRB protocols for research on human subjects (ClinicalTrials registration ID: NCT00752076). Animal experiment protocols are approved by Institutional Animal Care and Utilization Committee (IACUC 14-02-647, Academia Sinica).

### Statistics

The statistic significances of the experimental results were assessed by Independent-Samples *T*-Test (Two-tailed) or Paired *T*-Test (for clinical samples containing paired tumor and normal parts) using SigmaPlot v.12 (Systat Software Inc.). *P* < 0.05 is considered significant.

## SUPPLEMENTARY MATERIALS FIGURES AND TABLES


