# Supplementary material for: Hedgehog pathway maintains cell survival under stress conditions, and drives drug resistance in lung adenocarcinoma
Source: Oncotarget. 2016 Mar 22;7(17):24179–93. doi: 10.18632/oncotarget.8253 (PMC5029693; doi:10.18632/oncotarget.8253)
Supplement: Supplementary file 1 [file oncotarget-07-24179-s001.pdf]

Hedgehog pathway maintains cell survival under stress conditions, and drives drug resistance in lung adenocarcinoma

Supplementary Materials

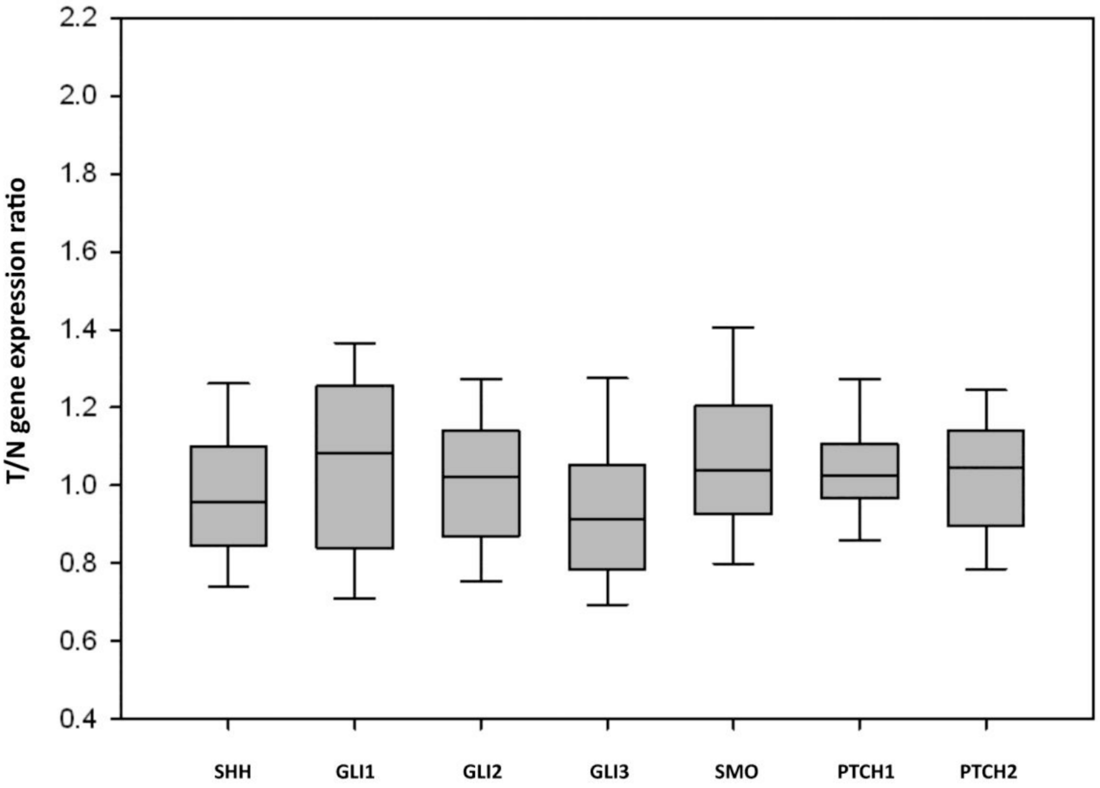

Supplementary Figure S1: The relative gene expressions of HH pathway components between tumor and normal parts in lung adenocarcinoma (LAC) patients samples. The microarray data set (GSE10072) containing information of both tumor and adjacent normal tissue from 49 patient samples were analyzed for T/N ratio (tumor vs. normal part) of gene expressions of HH pathway components. Because of the older chip version (Affimatrix HG-U133A) applied in the analysis of these samples, HHIP gene was not included.

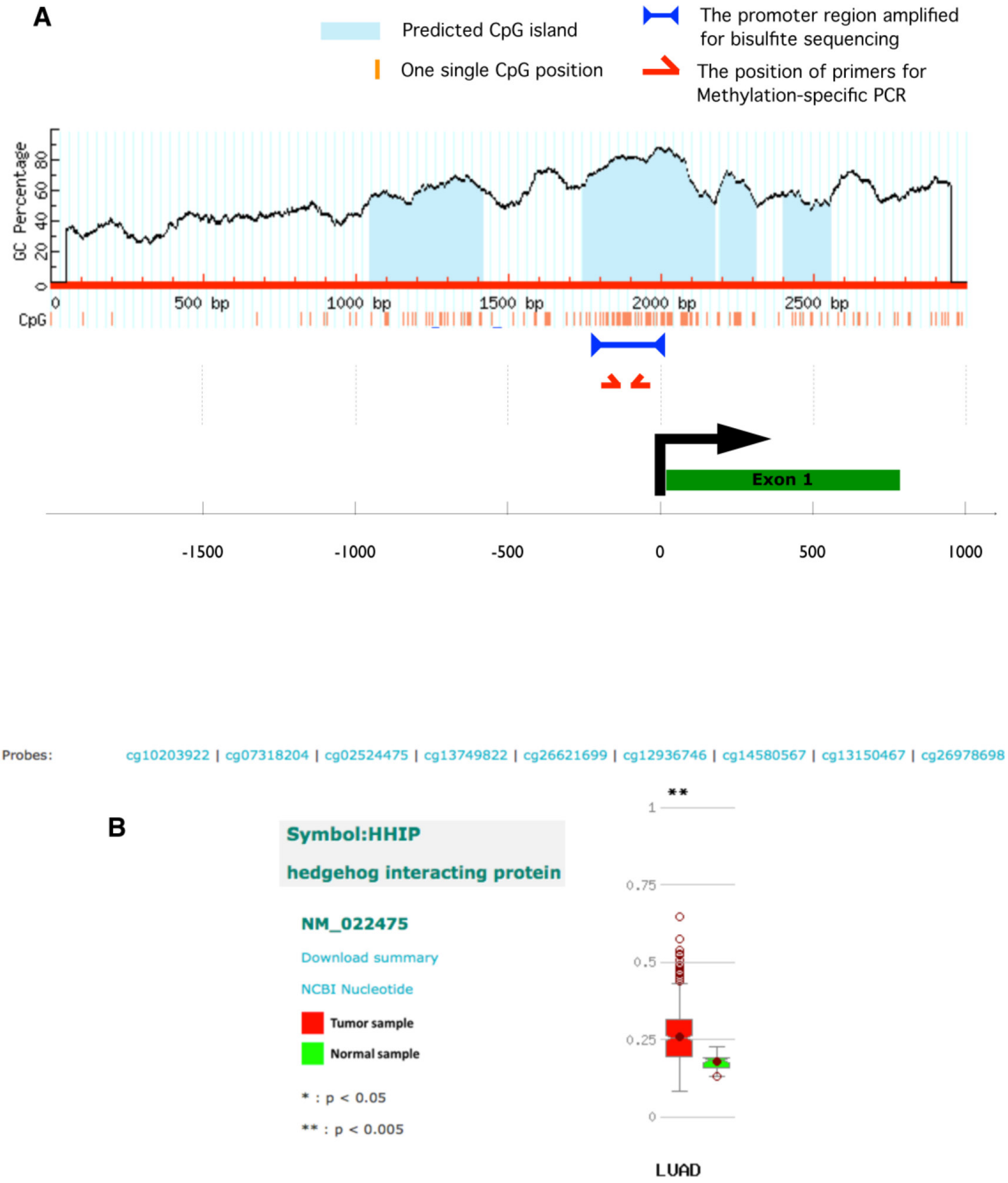
A
Predicted CpG island
One single CpG position
The promoter region amplified for bisulfite sequencing
The position of primers for Methylation-specific PCR
GC Percentage
0
20
40
60
80
0
500 bp
1000 bp
1500 bp
2000 bp
2500 bp
CpG
Exon 1
-1500
-1000
-500
0
500
1000
Probes:
cg10203922 | cg07318204 | cg02524475 | cg13749822 | cg26621699 | cg12936746 | cg14580567 | cg13150467 | cg26978698
B
Symbol:HHIP
hedgehog interacting protein
NM_022475
Download summary
NCBI Nucleotide
Tumor sample
Normal sample
* : p < 0.05
** : p < 0.005
**
1
0.75
0.5
0.25
0
LUAD

C

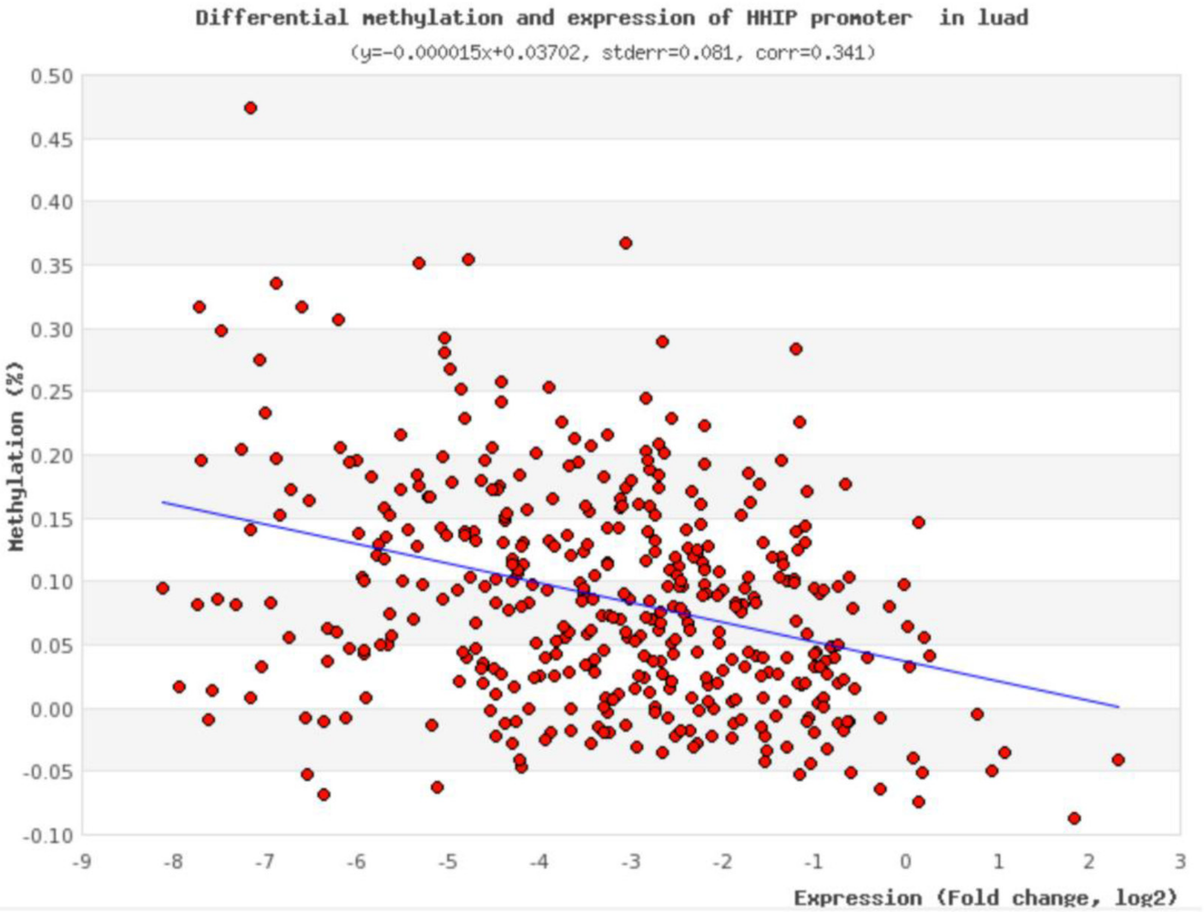

**correlation:**	-0.19597676837781
**P value:**	0
**regression:**	mx+b: m=0.067020690630642, b=-2.0572210898209E-5

Supplementary Figure S2: The methylation analysis of HHIP promoter in LAC cell lines and tumor samples. (**A**) HHIP promoter sequence was obtained using Transcriptional Regulatory Element Database (https://cb.utdallas.edu/cgi-bin/TRED/tred.cgi?process = searchPromForm, Michael Zhang Lab, Cold Spring Harbor Laboratory). The CG island distribution was analyzed using MethPrimer (http://www.urogene.org/methprimer/, The Li Lab, Department of Urology, UCSF). The Figure was generated and modified from MethPrimer. The Bisulfite Sequencing (BS)-specific primers were designed according to a previous publication[1] (Supplementary Table S2), and the relative location to be amplified was indicated as blue inverted arrows. The Methylation-Specific PCR (MSP) primers were designed according to a previous publication[2] (Supplementary Table S2), and the primer location was indicated as red arrows. The thermo-cycle of PCR is presented on Supplementary Table S3. The methylation status of HHIP promoter (**B**), and the correlation between methylation and gene expression levels (**C**) were analyzed in 492 LAC patient samples from TCGA open data base. The analysis was performed on the website of Methylation Data Base (http://methhc.mbc.nctu.edu.tw/php/index.php, National Chiao Tung University, Hsinchu, Taiwan).

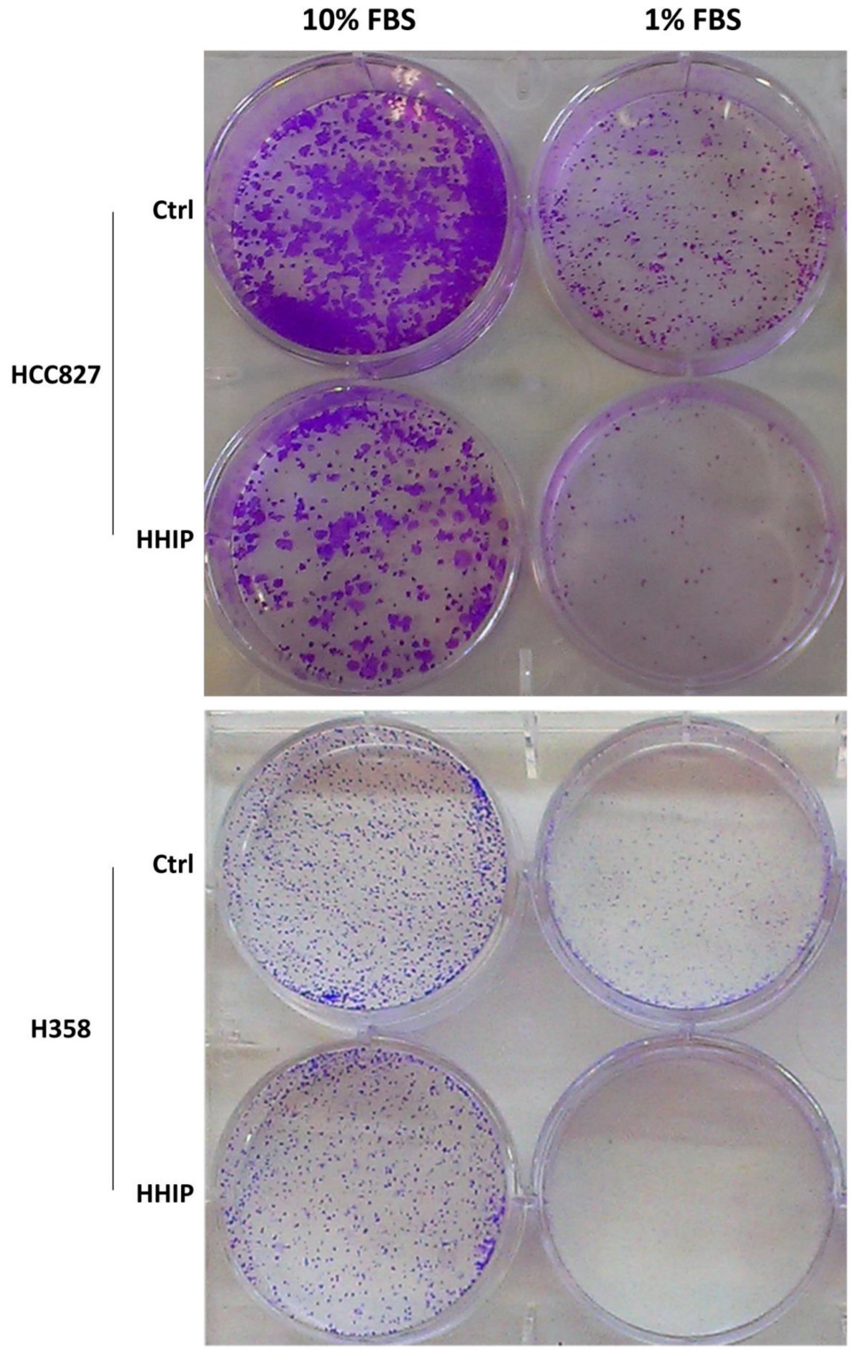

Supplementary Figure S3: The clonogenicity of LAC cells overexpressing HHIP. The original image of that presented on Figure 3.

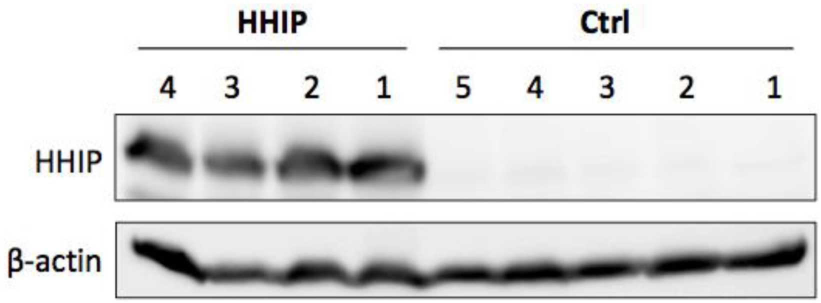

Supplementary Figure S4: The HHIP overexpression in HCC827 tumors. The lysates from HCC827 subcutaneous tumors (Figure 3E–G) overexpressing HHIP or control were verified for HHIP overexpression using Western-Blot. Each Number indicates an individual tumor.

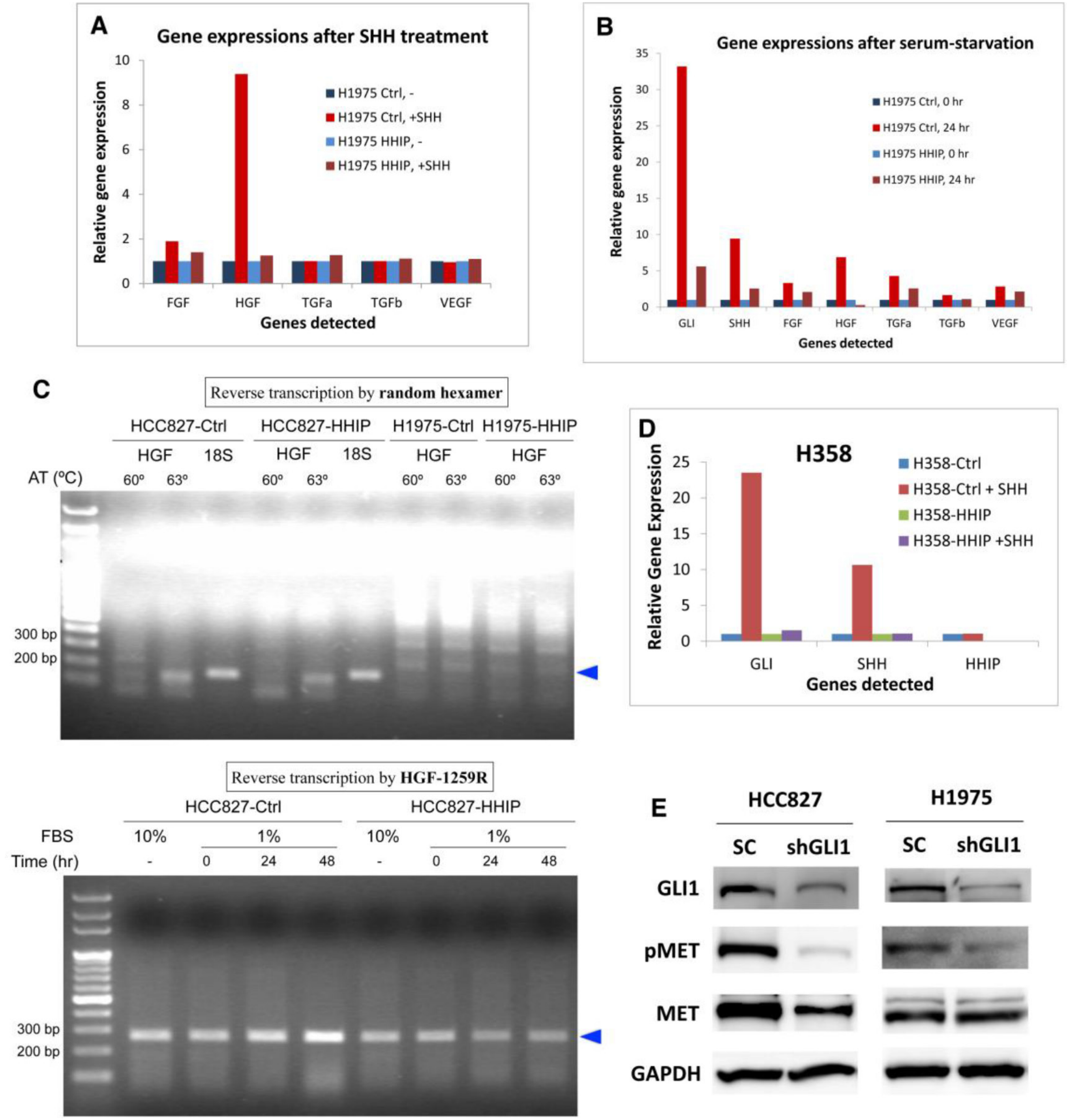

Supplementary Figure S5: The gene expression analysis of different growth factors in LAC cells. (**A**) H1975 cells overexpressing HHIP or RFP as control (Ctrl) were treated with SHH, and analyzed for gene expressions of a series of growth factors including HGF. (**B**) H1975 cells overexpressing HHIP or RFP were serum-starved for 24 hr, and analyzed for gene expressions of a series of growth factors. (**C**) Because the qPCR analysis of HGF gene expression frequently showed instable results when reproducing the experiment, the PCR products were investigated using agarose gel-electrophoresis. The results showed that numerous non-specific bands were frequently produced when the random hexamer (provided by the commercial kit of reverse transcription) was applied, despite of different annealing temperatures (AT) tested (upper figure). To address the problem, we used an HGF mRNA specific primer HGF-1259R for reverse transcription, which remarkably reduced the non-specific bands (lower figure). The primer pair used for HGF mRNA detection was HGF-739F/HGF-964R (Supplementary Table S2), with an predictive product of 270 bp as indicated on the Figure (blue triangle). (**D**) The gene expressions of GLI1, SHH, and HHIP were detected using qPCR in H358 cells overexpressing HHIP or RFP (Ctrl), after treatment with SHH. (**E**) The protein levels of GLI1, pMET, and total MET were analyzed in HCC827 and H1975 cells expressing shRNA against GLI1 (shGLI1) or scramble shRNA (SC).

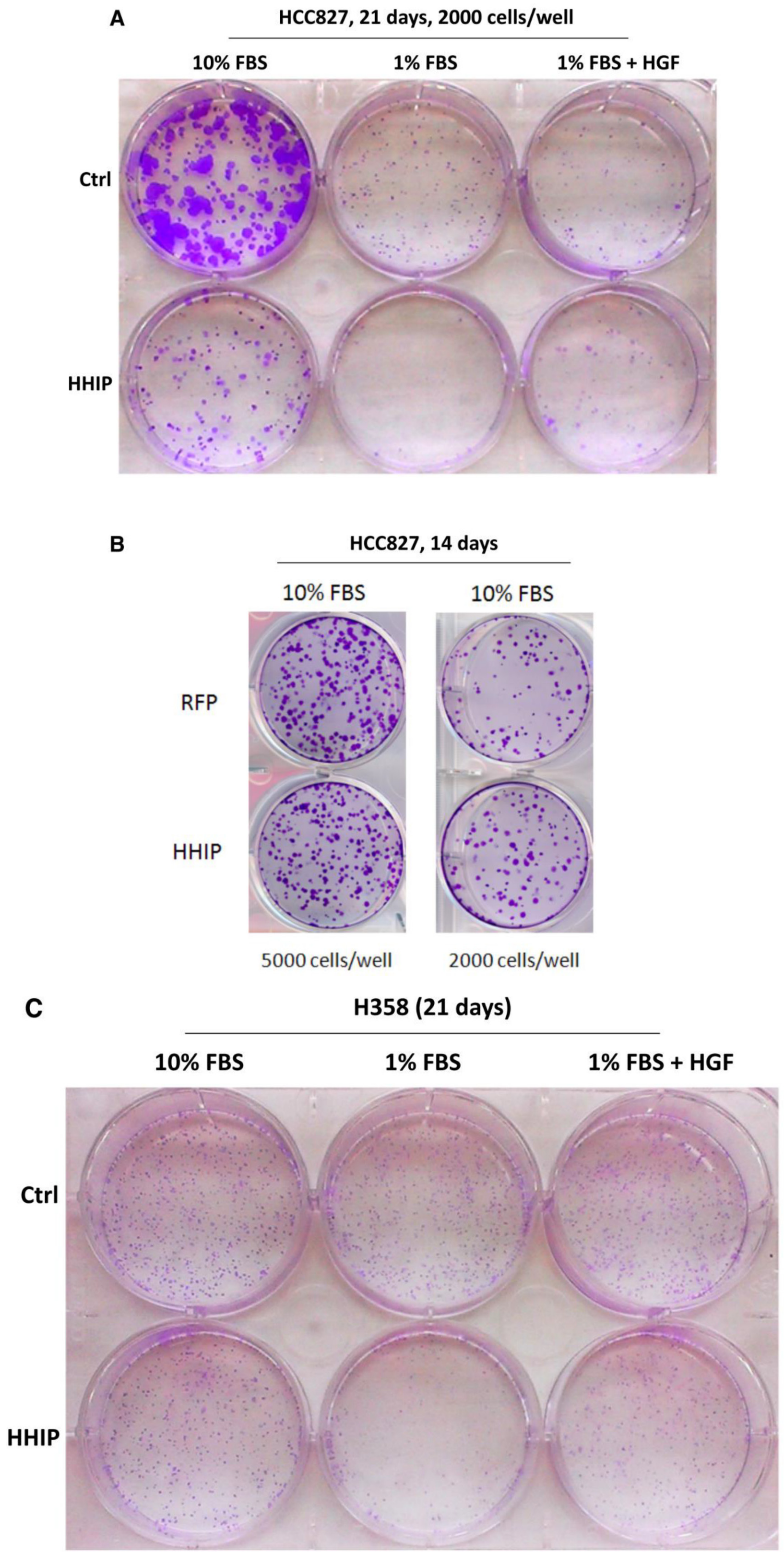

Supplementary Figure S6: The effect of HGF on clonogenicity of LAC cells overexpressing HHIP. (**A**) The colony formation of HCC827 cells overexpressing HHIP or control in 10% or 1% FBS, with or without HGF treatment, for 21 days. HCC827 control group in 10% FBS showed overgrowth in this time course. (**B**) The colony formation of HCC827 cells overexpressing HHIP or control in 10% FBS for 14 days. Both groups showed no overgrowth, and corresponding colony numbers. (**C**) The colony formation of H358 cells overexpressing HHIP or control in 10% or 1% FBS, with or without HGF treatment, for 21 days.

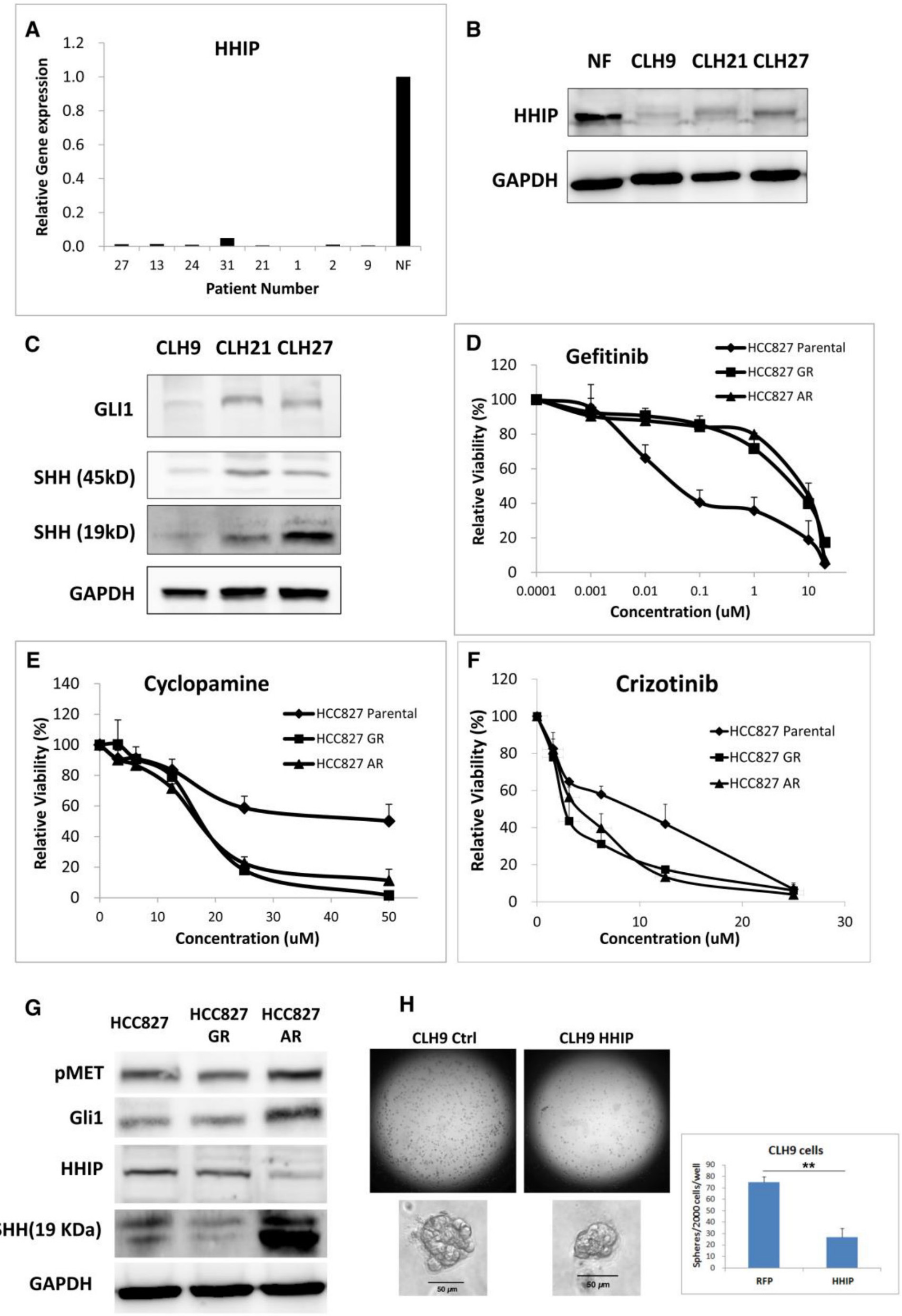

Supplementary Figure S7: The drug sensitivity and HH activity in EGFR-TKI sensitive or resistant cells. (**A**) The tumor samples obtained from LAC patients relapsed from EGFR-TKI treatment (Supplementary Table S1) were analyzed for HHIP gene expression using qPCR. The HHIP expression in normal fibroblast (NF) obtained from normal tissue of patient was presented as control. (**B**) NF and a part of primary cultured tumor cells were analyzed for HHIP protein expressions using Western-Blot. (**C**) Primary cultured tumor cells were analyzed for GLI1 and SHH protein expressions using Western-Blot. 45-kD precursor and 19-kD active form of SHH were both presented. (**D–F**), HCC827-GR (Gefitinib-resistant) and HCC827-AR (Afatinib-resistant) cell lines were generated by long-term treatment with increasing doses of TKI on HCC827 cells for 60 days. For Gefitinib: 0.2 μM for 10 days, 2 μM for 10 days, 5 μM for 10 days, and 10 μM for 30 days. For Afatinib (Santa Cruz): 0.01 uM for 10 days, 0.1 uM for 20 days, 0.5 uM for 30 days. After 60 days, the resistant cells were maintained in culture medium without EGFR-TKI. EGFR T790M secondary mutation or MET amplification were not observed in these resistant cells (data not shown). HCC827 parental, -GR, and -AR cells were tested for survival curve against (D) Gefitinib (EGFR-TKI), (E) Cyclopamine (HH inhibitor), or (F) Crizotinib (MET inhibitor). (**G**) The endogenous protein levels of pMET, GLI1, HHIP, and SHH were analyzed using western-blot in HCC827 parental, GR, and AR cells. (**H**) CLH9 cells overexpressing HHIP or control were analyzed for spheroid formation in serum-free 3D matrix, and quantified. $^{**}P < 0.01$, $n = 6$.

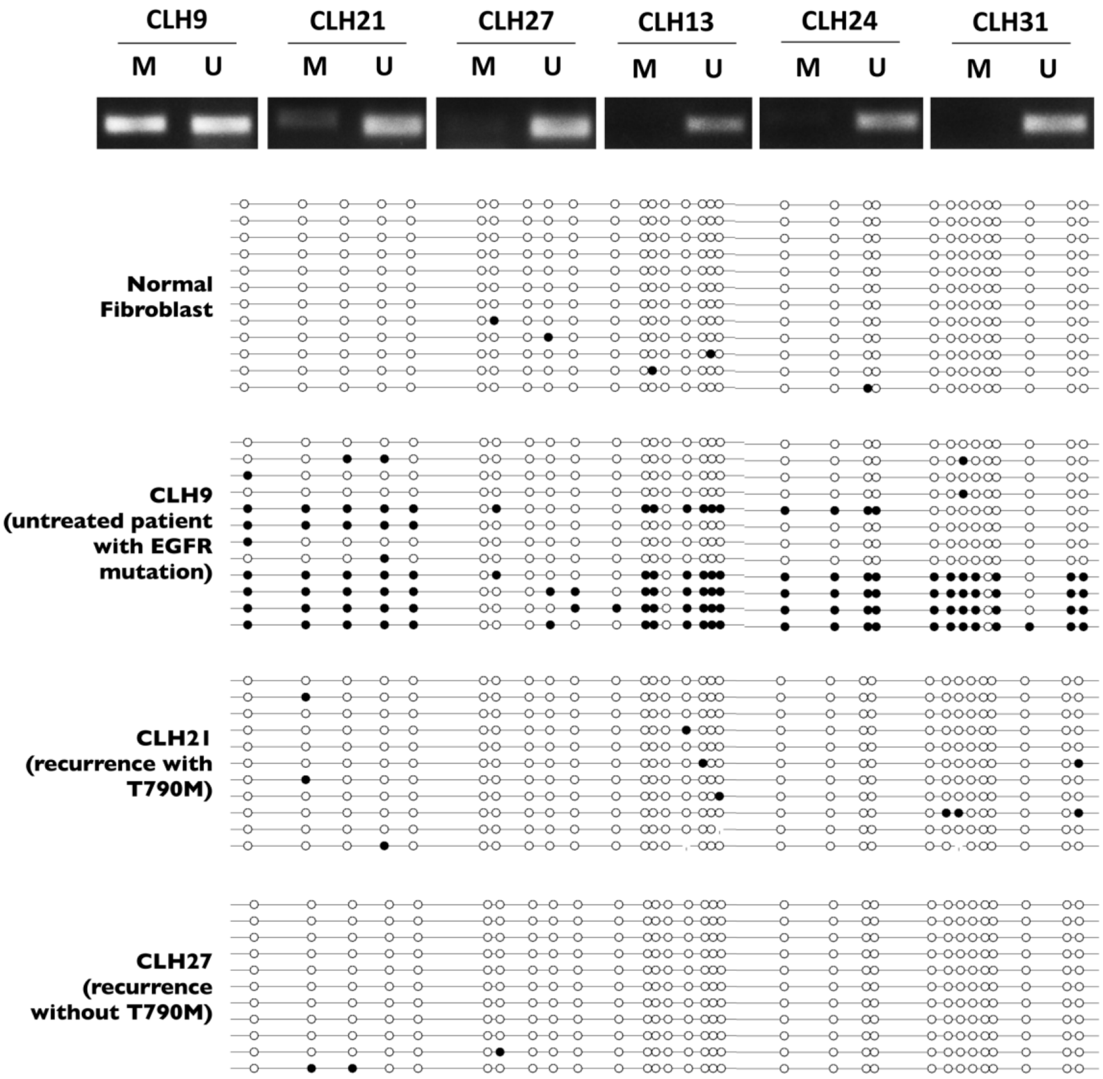

Supplementary Figure S8: The methylation analysis of HHIP promoter in primary LAC tumor cells with acquired resistance to EGFR-TKI. The BS and MSP analyses, as described on Supplementary Figure S2, were performed in primary cell lines and normal fibroblast. The solid circle indicates a methylated CG site, while empty circle unmethylated.

Supplementary Table S1: The LAC tumor samples from patients that relapsed from EGFR-TKI treatment

Nr.	Type	IC50 TKI	EGFR mutation	Patient state
CLH27	Adenocarcinoma	> 10 µM	Exon 19 deletion	
CLH13	Adenocarcinoma	> 10 µM	G7I9A	Responded to TKI; relapsed without T790M
CLII24	Adenocarcinoma	> 10 µM	L858R	
CLH31	Adenocarcinoma	> 10 µM	L858R	
CLH21	Adenocarcinoma	> 10 µM	L858R+T790M	Responded to TKI; relpascd with T790M
CLH1	Adenocarcinoma	> 10 µM	WT + Q787Q + 1 Her2 insertion	No respond to TKI
CLH2	Poor differentiated	> 10 µM	L.858R + V834L	
CLH9	Adenocarcinoma	0.1–1 µM	Exon 19 deletion	Untreated

Supplementary Table S2: The primer list

Name	Sequence (5′ to 3′)
HHIP-F	CCAGCAAAGTCCTGTGACAA
HHIP-R	CCCACTAGTGCCGAGACAG
GLI-3038F	CTCCCGAAGGACAGGTATGTA AC
GL1-3261R	CCCTACTCTTTAGGCACTAGAGTTG
SHH-456F	GTAAGGACAAGTTGAACGCTTTG
SHH-694R	GATATGTGCCTTGGACTCGTAGTA
TGFα-249F	ATGGTCCCCTCGGCTGGACAG
TG Fα-725 R	TCCTCCTCTGGGCTCTTCAG
TGFβ-1128F	TGTGTGCTGAAGCCATCGTTG
TGFβ-1337R	CCGGCTTGTCTGAAAAGGTCA
FGF-579F	GGCTTCTTCCTGCGCATCCA
FGF-911R	GCTCTTAGCAGACATTGGAAGA
HGF-1259R	CACCAGGGTGATTCAGACCC
HGF-739F	CGCTACGAAGTCTGTGACATTC
HGF-964R	CGGGTGTGAGGGTCAAGAGTATA
18S-F	GGCGGCGTTATTCCCATGA
18S-R	GAGGTTTCCCGTGTTGAG
GPPDH-F	GCATTGCCCTCAACGAC
GPPDH-R	GTCTCTCTCTTCCTCTTGTGC
HHIP-BS-F	GGGGAGGAGAGAGGAGTTTG
HUIP-BS-R	CCCRACRACCTCCCTACTA CTA
HHIP-MSP-Meth-F	GTAGTAGTCGGGT AGTTTCGG A ATTTTC
H H1P-M SP-Meth-R	AAAAACGACTAACCGCGACG
H HIP-M S P-non Meth-F	AGTAGTTGGGTAGTTTTGGAATTTTTGG
HHlP-MSP-non.Meth-R	AAAAACAACTAACCACAACA

Supplementary Table S3: The thermo-cycle program of PCR

PCR program for Bisulfite Sequencing

Primer pair: HHIP-BS-F and HHIP-BS-R Thermocycle program:

Step	Temp. (°C)	Duration	Repeat
1	95	5 min	
2	95	10 sec	
3	59	30 sec	
4	72	10 sec	To step 2 for 40 cycles
5	72	10 min	

PCR program for Methylation-Specific PCR

Primer pair: HHIP-BS-F and HHIP-BS-R Thermocycle program:

Step	Temp. (°C)	Duration	Repeat
1	95	5 min	
2	95	30 sec	
3	60	40 sec	
4	72	50 sec	To step 2 for 40 cycles
5	72	10 min	

PCR program for amplification of HGF cDNA

Primer pair: HGF-739F and HGF-964R Thermocycle program:

Step	Temp. (°C)	Duration Repeat	
1	95	5 min	
2	95	5 sec	
3	61	15 sec	
4	72	10 sec	To step 2 for 35 cycles
5	72	10 min	

REFERENCE

1 Song Y, Tian Y, Zuo Y, Tu JC, Feng YF, Qu CJ. Altered expression of PTCH and HHIP in gastric cancer through their gene promoter methylation: novel targets for gastric cancer. Mol Med Rep. 2013; 7:1159–1168.

2 Song Y, Zuo Y. Occurrence of HHIP gene CpG island methylation in gastric cancer. Oncology letters. 2014; 8:2340–2344.
